# The Impact of Early Postoperative Urinary Incontinence on Presenteeism After Robot‐Assisted Radical Prostatectomy for Prostate Cancer: A Prospective Cohort Study

**DOI:** 10.1111/luts.70055

**Published:** 2026-03-18

**Authors:** Noriko Nakayama, Tetsuya Tsuji, Akira Kumagai

**Affiliations:** ^1^ Department of Rehabilitation Teine Keijinkai Hospital Sapporo Hokkaido Japan; ^2^ Department of Rehabilitation Medicine Keio University School of Medicine Tokyo Japan; ^3^ Miyanosawa Nephrology and Urology Clinic Sapporo Hokkaido Japan

**Keywords:** presenteeism, prostate cancer, quality of life, urinary incontinence

## Abstract

**Objectives:**

Urinary incontinence (UI) in the first 3 months after robot‐assisted radical prostatectomy (RARP) frequently impairs work performance, yet its quantitative impact on presenteeism remains unclear. This prospective cohort study longitudinally evaluated how early postoperative UI influences presenteeism among employed Japanese men undergoing RARP.

**Methods:**

We consecutively enrolled 92 employed male patients scheduled for RARP and assessed them preoperatively, at discharge, and at 1‐ (PS‐1) and 3‐month (PS‐3) post‐surgery. Outcomes were the International Consultation on Incontinence Questionnaire‐Short Form (ICIQ‐SF), WHO Health and Work Performance Questionnaire (HPQ)‐Presenteeism Scale, King's Health Questionnaire (KHQ), and Kessler Psychological Distress Scale (K6).

**Results:**

Of the 92 eligible participants, 85 (92.4%) completed the PS‐1 and 80 (87.0%) completed the PS‐3 assessment. Median ICIQ‐SF total scores increased from 0 (IQR 0–2) preoperatively to 9 (6–12) at PS‐1, remaining elevated at 7 (4–10) at PS‐3 (*p* < 0.001). Mean HPQ‐presenteeism declined from 81.4% ± 13.9% preoperatively to 64.9% ± 18.8% at PS‐1, partially recovering to 75.3% ± 16.1% at PS‐3 (*p* < 0.001). At PS‐1, UI impact on daily life (*ρ* = −0.45) and ICIQ‐SF total (*ρ* = −0.43) were moderately associated with lower presenteeism; at PS‐3, correlations persisted and extended to multiple KHQ subscales (*ρ* = −0.41 to −0.53).

**Conclusions:**

Early postoperative UI after RARP produces a clinically meaningful reduction in work productivity, greatest at PS‐1 and still evident at PS‐3. Targeted continence care and occupational support during this window may mitigate productivity loss and facilitate sustainable return to work.

## 1 Introduction

Prostate cancer is now the most frequently diagnosed malignancy in Japanese men, with 87 756 new cases reported in 2020 [1] and an average annual increase of 1.3% [2]. Thanks to advances in detection and treatment, the five‐year relative survival rate reached 99.1% for the period from 2009 to 2011 [3], meaning that the majority of patients live long enough to confront survivorship issues while still in the workforce.

Robot‐assisted radical prostatectomy (RARP) offers excellent oncological control; however, postoperative urinary incontinence (UI) has been reported to affect 59.4%–87% [4, 5] of patients, depending on the definition. Although UI usually improves within 1 year [6, 7], the peak symptom burden consistently occurs at 1 month, when the International Consultation on Incontinence Questionnaire‐Short Form (ICIQ‐SF) total score is highest. During this interval, patients may experience discomfort associated with UI, need to change urinary pads, and in some cases may even be forced to change clothes, which significantly reduces patient quality of life (QOL) [8] and delays return to work.

Presenteeism—defined by the World Health Organization (WHO) as when employees are physically present at work but are not fully functioning due to health issues, stress, or other personal problems [9]—has emerged as a critical but under‐studied dimension of cancer survivorship. Although there have been cross‐sectional studies on postoperative work ability and presenteeism [10, 11], there have not been any reports on changes in presenteeism over time in the early stages after prostate cancer surgery, and the relationship between UI and presenteeism remains unclear.

Accordingly, we conducted a single‐center prospective cohort study to quantify changes in presenteeism from before surgery to 3 months after RARP and to identify UI‐related determinants of productivity loss. By clarifying these relationships, we aim to inform timely continence management that facilitate early, effective work reintegration.

## 2 Methods

### 2.1 Study Design and Participants

This prospective cohort study surveyed 92 employed Japanese male patients diagnosed with prostate cancer who underwent RARP at Teine Keijinkai Hospital between June 2021 and February 2024. Inclusion required paid employment ≥ 20 h week^−1^ at admission. Exclusion criteria were (i) an inability to understand the questionnaire survey, (ii) cognitive decline (Mini Mental State Examination: MMSE ≤ 21), and (iii) an inability to obtain consent.

Questionnaires were administered preoperatively (Preop), at discharge, and by post at 1 (PS‐1) and 3 months (PS‐3) after surgery; QOL and presenteeism instruments were not applied at discharge. The pelvic floor muscle training (PFMT) in this study consisted of a program presented using individual verbal instruction by a physiotherapist and educational leaflets, without the use of special biofeedback or electrical stimulation devices. The patients were admitted to the hospital the day before surgery, and on the day of admission, an educational leaflet was used along with anatomical atlases to explain the function of the pelvic floor muscles. With the patients in the supine or lateral position, the therapist palpated or the patient's own digital examination confirmed that the anus and perineum were being pulled inward and toward the head. As compensatory movements, patients were instructed to avoid excessive contraction of the rectus abdominis, gluteal, and adductor muscles, as well as breath‐holding. Training consisted of a combination of “fast contraction (repeated contraction and relaxation at maximum contraction)” and “sustained contraction (moderate sustained contraction)” in various positions, including supine, sitting, and standing. The next day after surgery, the patients began training, focusing on getting out of bed. If there was no pain around the bladder catheter insertion site, PFMT was gradually started. After the bladder catheter was removed on the fifth day after surgery, the patients practiced PFMT tailored to their individual activities of daily living and work. For example, one patient practiced lifting movements while contracting their pelvic floor muscles, climbing stairs, and shoveling snow, and was discharged on the sixth day after surgery.

### 2.2 Outcome Measures

We reviewed medical records to extract data on the following evaluation items: age at time of surgery, obesity level (body mass index: BMI value), hospitalization days, period from diagnosis to surgery, pathological T stage (pT) as assessed in accordance with the Union for International Cancer Control (UICC) TNM classification, eighth edition, the International Society of Urological Pathology (ISUP) Grade Group system, blood data at the time of hospitalization (Hb value, Alb value, TP value), whether or not the patient had received pre‐post operative hormone therapy, surgical procedure (whether or not nerve sparing was performed, whether or not lymph node dissection was performed), intraoperative findings (blood loss, operation time), and duration of bladder catheterization post‐surgery (days).

The questionnaire also included the number of pads used per day and pad‐free rate (the pad‐free rate was defined as zero pads used per day) at PS‐1 and PS‐3, work‐related questions about employment status and employment form (regular/non‐regular employment, normal work/restrictions on work [reduction of working hours, restrictions on overtime work, change of work location, reduction of late‐night work, shift to daytime work]/leave of absence), and whether they worked for a large or small/medium‐sized employer. We also asked about the time it took to get from the work station to the toilet, which was included as part of the survey on the urination environment during work.

The Japanese version of the ICIQ‐SF is a self‐administered questionnaire evaluation tool for the determination of UI classification (stress, urge, mixed, overflow) and severity [12, 13]. Question 1 covers frequency of UI, Question 2 covers volume of UI, and Question 3 deals with the impact that incontinence has on daily life. The total score is 21, with a higher score indicative of more severe symptoms [14]. In this study, evaluations were performed before surgery, at discharge from hospital, and at PS‐1 and PS‐3.

The Japanese version of the Health and Work Performance Questionnaire (WHO‐HPQ) [15, 16] is a questionnaire that quantitatively evaluates presenteeism and absenteeism (sickness absence). In this study, we only evaluated pre‐ and postoperative presenteeism and did not go as far as to calculate the loss costs due to health problems. Furthermore, answering all items would be a heavy burden on patients and could have led to a low response rate, so questions regarding working hours (per day and per month) were excluded, with questions B10 and B11 selected for use. Each question was scored from 0 (worst performance) to 10 (best performance), and displayed as a percentage from 0 to 100%. A higher score indicates higher productivity (i.e., lower presenteeism).

The Japanese version of the King's Health Questionnaire (KHQ) [17] is a health‐related QOL assessment scale designed to measure the impact of urinary symptoms on QOL. It consists of nine subscales, each of which is scored from 0 to 100, with a higher score indicating a lower QOL.

The Japanese version of the Kessler Psychological Distress Scale (K6) [18] was used as an assessment tool to screen for symptoms of psychological distress, such as depression and anxiety disorders. It consists of six questions, each of which is scored from 0 to 4 points, for a total score of 0 to 24 points, with higher scores reflecting more severe psychological distress. A score of 13 or more was considered to indicate severe psychological distress.

### 2.3 Sample‐Size Calculation

Using G*Power 3.1 (effect size 0.40 [as there is no previous research, we used the criteria proposed by Cohen as a reference], *α* = 0.05, power = 0.80) for repeated‐measures ANOVA, 76 participants were required; we therefore recruited 92 patients to compensate for attrition.

### 2.4 Statistical Analysis

The Friedman test was used to analyze changes over time in the frequency of UI, volume of UI, and the impact of UI on daily life (based on the ICIQ‐SF) from Preop to PS‐3. If significant differences were observed, multiple comparisons were performed using the Wilcoxon‐Nemenyi–McDonald–Thompson test. One‐way analysis of variance was used for the ICIQ‐SF total score and the K6, KHQ, and WHO‐HPQ scores. If significant differences were found, Tukey's test was performed as a post hoc test. Correlations between presenteeism and each evaluation item were examined using Pearson's correlation coefficient and Spearman's rank correlation. Additionally, to analyze the impact of presenteeism on employment status, employment type (regular/non‐regular employment) and working style (normal work/restrictions on work) were classified into two groups and Student's *t*‐test was used for analysis. The time from the work station to the toilet was classified into three groups, and presenteeism at PS‐1 and PS‐3 was compared using ANOVA. Significance was set at *p* < 0.05. Analyses were performed using JMP Pro 18 (SAS Institute Inc., Cary, NC).

## 3 Results

### 3.1 Participant Flow and Follow‐Up

**FIGURE 1:**
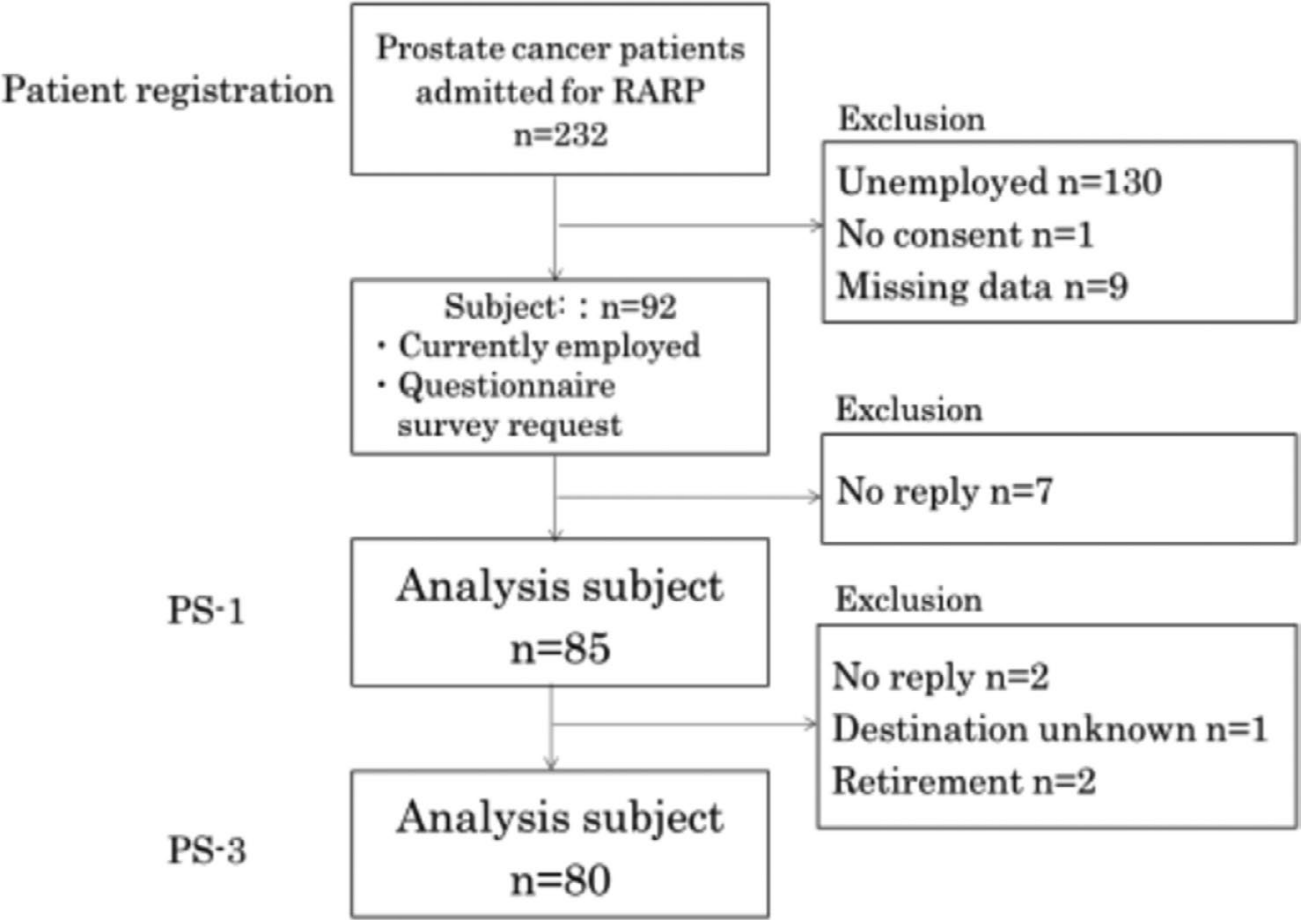
Flowchart of subject enrollment. A total of 232 men underwent RARP between June 2021 and February 2024; 92 met the eligibility criteria and consented, 85 (92.4%) returned the 1‐month questionnaire, and 80 (87.0%) completed 3‐month follow‐up.

A total of 232 patients underwent RARP during the study period; 92 met all eligibility criteria and consented to participate. Questionnaires were returned by 85 (92.4%) at PS‐1 and 80 (87.0%) at PS‐3. No serious adverse events were reported (Figure 1). Five participants (5.9%) were lost between PS‐1 and PS‐3; analyses therefore used complete‐case data at each time point. Sensitivity analyses with last‐observation‐carried‐forward yielded comparable results (data not shown).

### 3.2 Baseline Characteristics

Baseline demographic and clinical information are summarized in Table 1. The mean age was 65.6 ± 6.7 years. The pathological T stage (pT) was pT1 in two patients (2.2%), pT2 in 46 patients (51.1%), pT3 in 39 patients (43.3%), and pT4 in three patients (3.3%). Median catheterization and hospital stay were 5 days (IQR 5–5) and 7 days (IQR 7–7.3), respectively. The average number of pads used per day at PS‐1 and PS‐3 was 3.1 ± 2.8 and 1.9 ± 1.9, respectively, and the pad‐free rate was 6.7% and 13.4%, respectively.

**TABLE 1:** Baseline demographic and clinical characteristics of the participants.

Age at surgery (years) mean ± SD (min–max)	65.6 ± 6.7 (45–76)
Hospitalization days (days) median (IQR)	7.0 (7.0–7.3)
BMI (kg/m^2^)	24.6 ± 3.1 (16.6–33.1)
Period from diagnosis to surgery (days)	104.3 ± 132.3 (24.0–968.0)
Duration of bladder catheterization postop (days) median (IQR)	5.0 (5.0–5.0)
Pathological T classification for prostate cancer, *n* (%)	
pT1	2 (2.2)
pT2	46 (51.1)
pT3	39 (43.3)
pT4	3 (3.3)
Unclear	2
Grade group, *n* (%)	
1	20 (21.7)
2	28 (30.4)
3	13 (14.1)
4	18 (19.6)
5	13 (14.1)
Preop hormone therapy, *n* (%)	9 (9.8)
Postop hormone therapy, *n* (%)	12 (13.3)
Postop radiation therapy, *n* (%)	0 (0.0)
Intraoperative findings	
Amount of bleeding (mL) (min–max)	91.5 ± 97.9 (5‐400)
Operative time (min)	200.5 ± 49.6 (114‐327)
Lymph node dissection, *n* (%)	61 (71.8) missing data 1
Nerve sparing, *n* (%)	29 (34.1) missing data 1

### 3.3 Employment Status

Most participants were regular, unrestricted employees (77.6% regular; 89.5% in normal duties); two‐thirds worked for small/medium‐sized enterprises (64.5%), and 72.5% could reach a toilet within 5 min of their workstation (Table 2).

**TABLE 2:** Employment status and workplace environment at baseline (regular vs. non‐regular employment, job restrictions, company size, and toilet accessibility).

Form of employment	
Regular employees, *n* (%)	59 (77.6)
Unclear	9
Working style	
Normal work, *n* (%)	68 (89.5)
Employment restriction, *n* (%)	8 (10.5)
Short working hours, *n* (%)	6 (7.9)
Over‐time work restriction, *n* (%)	1 (1.3)
Work place restriction, *n* (%)	1 (1.3)
Reduction of late‐night work, *n* (%)	0 (0.0)
Change to daytime work, *n* (%)	0 (0.0)
Unclear	9
Employer scale	
Large enterprise, *n* (%)	18 (23.7)
Small/medium‐sized employers, *n* (%)	49 (64.5)
Unclear	9
The toilet environment at the work station	
The toilet within 5 min, *n* (%)	58 (72.5)
5‐10 min, *n* (%)	16 (20.0)
10 min or more *n* (%)	6 (7.5)
Unclear	5
Number of pads used (pads per day)	
PS‐1	3.1 ± 2.8
PS‐3	1.9 ± 1.9
Pad‐free rate *n* (%)	
PS‐1	5 (6.7)
PS‐3	9 (13.4)

### 3.4 Urinary Incontinence Trajectory

**FIGURE 2:**
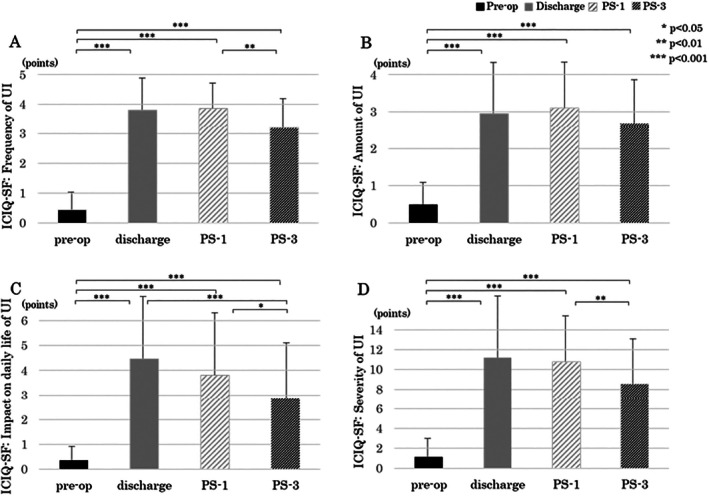
Changes in International Consultation on Incontinence Questionnaire‐Short Changes in ICIQ‐SF scores. Form (ICIQ‐SF) scores. (A) Frequency of UI, (B) amount of UI, (C) impact of UI on daily life, and (D) ICIQ‐SF total score. Preop = before surgery; PS‐1 = 1 month after surgery; PS‐3 = 3 months after surgery.

ICIQ‐SF total score increased from 0 (IQR 0–2) Preop to 9 (6–12) at PS‐1 (*p* < 0.0001) and improved to 7 (4–10) at PS‐3 (*p* = 0.002 vs. PS‐1). All individual item scores followed the same pattern, showing the greatest symptom burden at PS‐1 and partial recovery by PS‐3 (Figure 2).

### 3.5 Presenteeism Over Time

**FIGURE 3:**
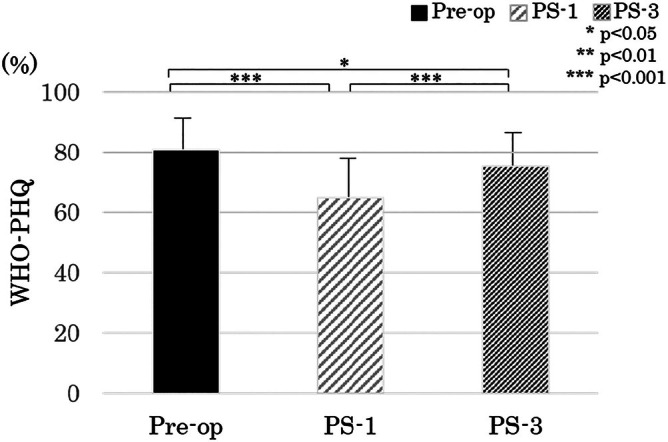
Changes in World Health Organization Health and Work Performance Questionnaire (WHO‐HPQ) presenteeism scores. Preop = before surgery; PS‐1 = 1 month after surgery; PS‐3 = 3 months after surgery.

Mean WHO‐HPQ presenteeism declined from 81.4% ± 13.9% Preop to 64.9% ± 18.8% at PS‐1 (*p* < 0.0001) and thereafter rebounded to 75.3% ± 16.1% at PS‐3 (*p* = 0.0003 vs. PS‐1; *p* = 0.048 vs. Preop) (Figure 3).

### 3.6 Associations Between UI Severity and Work Productivity

At PS‐1, presenteeism correlated moderately with ICIQ‐SF impact on daily life (*ρ* = −0.45), ICIQ‐SF total score (*ρ* = −0.43), and the KHQ Social limitations subscale (*ρ* = −0.42) (all *p* < 0.0001). These correlations persisted and strengthened at PS‐3, extending to multiple KHQ domains (e.g., General Health Perceptions *ρ* = −0.53). No significant correlation was found with psychological distress (Table 3).

**TABLE 3:** Correlations between WHO‐PHQ and other items.

WHO‐HPQ	Other items	Correlation coefficient	*p*
PS‐1	Impact of UI on daily life (ICIQ‐SF)	−0.45	< 0.0001
	ICIQ‐SF total scores	−0.43	< 0.0001
	General health perceptions (KHQ)	−0.27	0.0166
	Incontinence impact (KHQ)	−0.28	0.0125
	Role limitations (KHQ)	−0.37	0.0007
	Physical limitations (KHQ)	−0.24	0.0371
	Social limitations (KHQ)	−0.42	0.0001
	Personal relationships (KHQ)	−0.04	0.6991
	Emotions (KHQ)	−0.34	0.0037
	Sleep/energy (KHQ)	−0.23	0.0392
	K6	−0.26	0.0215
PS‐3	Impact of UI on daily life (ICIQ‐SF)	−0.41	0.0002
	ICIQ‐SF total scores	−0.42	0.0002
	General health perceptions (KHQ)	−0.53	< 0.0001
	Incontinence Impact (KHQ)	−0.38	0.0004
	Role limitations (KHQ)	−0.47	< 0.0001
	Physical limitations (KHQ)	−0.47	< 0.0001
	Social limitations (KHQ)	−0.42	0.0001
	Emotions (KHQ)	−0.50	< 0.0001
	Sleep/energy (KHQ)	−0.37	0.0007
	K6	−0.34	0.0026

*Note:* Spearman correlations between WHO‐HPQ presenteeism and UI, QOL, and psychological‐distress measures at PS‐1 and PS‐3 (ICIQ‐SF, KHQ subscales, K6).

### 3.7 The Impact of Postoperative Adjuvant Therapy and Presenteeism Due to Employment Status

**FIGURE 4:**
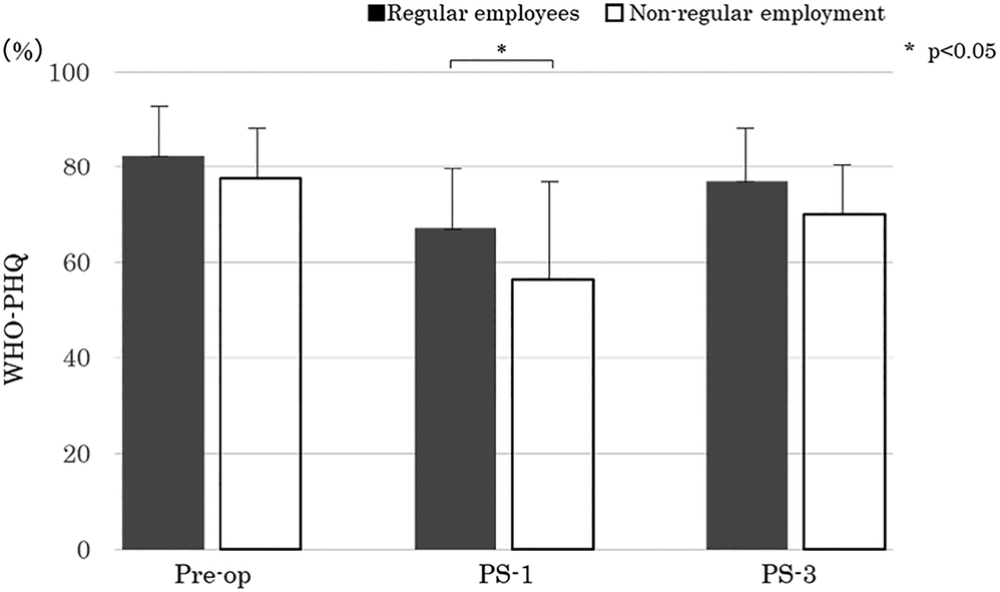
Comparison of presenteeism and working environment. Preop = before surgery; PS‐1 = 1 month after surgery; PS‐3 = 3 months after surgery.

Comparing presenteeism by employment status and work environment, non‐regular employees had lower work productivity than did regular employees at 1‐month post‐surgery (*p* = 0.038) (Figure 4). There were no significant differences in presenteeism according to work style (regular work/work restrictions) or time it took to get from the work station to the toilet.

### 3.8 Health‐Related QOL

**FIGURE 5:**
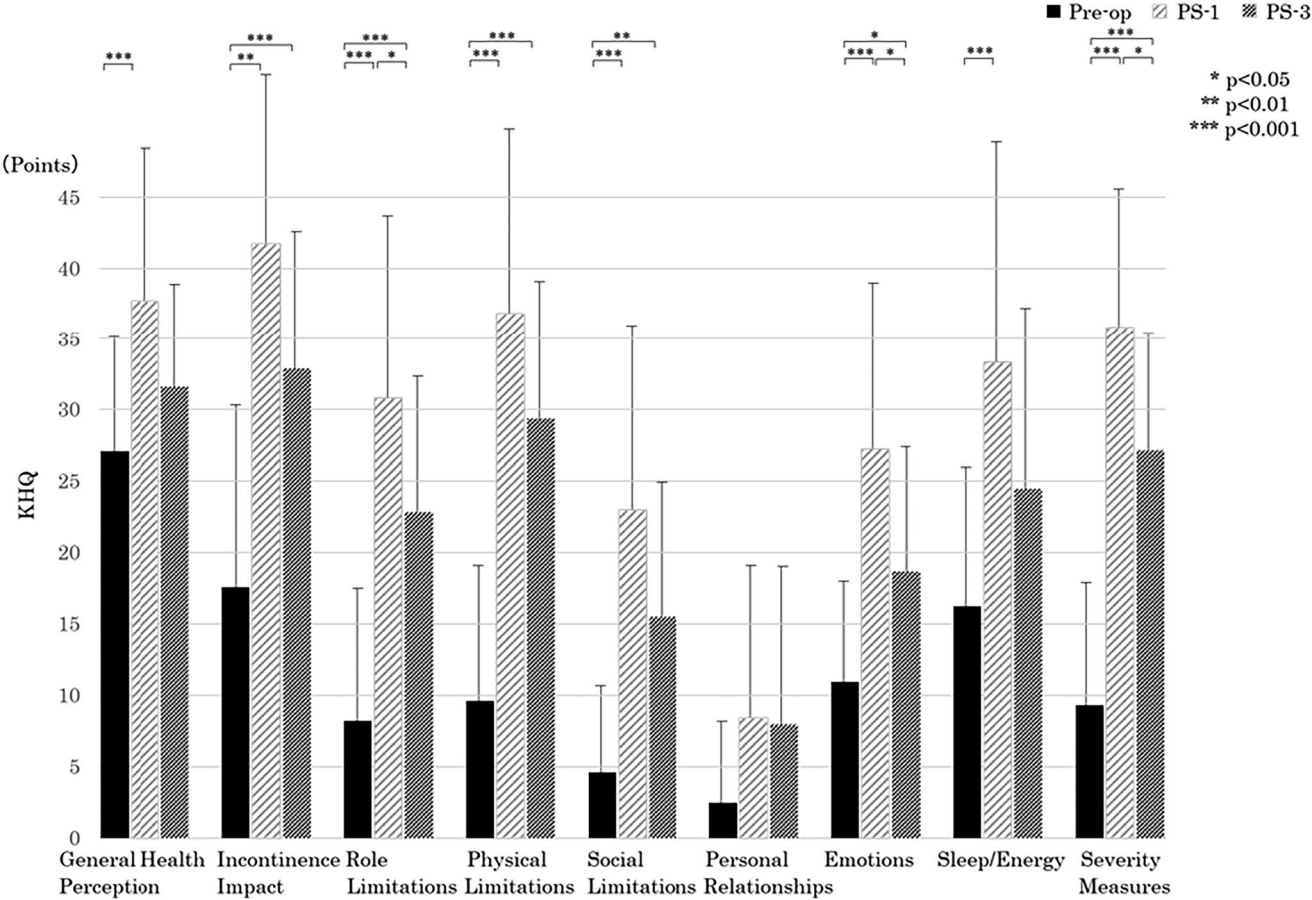
Changes in King's Health Questionnaire (KHQ) scores. Higher scores indicate worse health‐related quality of life. Preop = before surgery; PS‐1 = 1 month after surgery; PS‐3 = 3 months after surgery.

The KHQ composite score worsened significantly at PS‐1 and PS‐3 compared with preop (both *p* < 0.0001), but improved between PS‐1 and PS‐3 (*p* = 0.012). Subscales for Incontinence Impact, Role Limitations, and Physical Limitations remained higher (worse) at PS‐3 (Figure 5).

### 3.9 Psychological Distress

**FIGURE 6:**
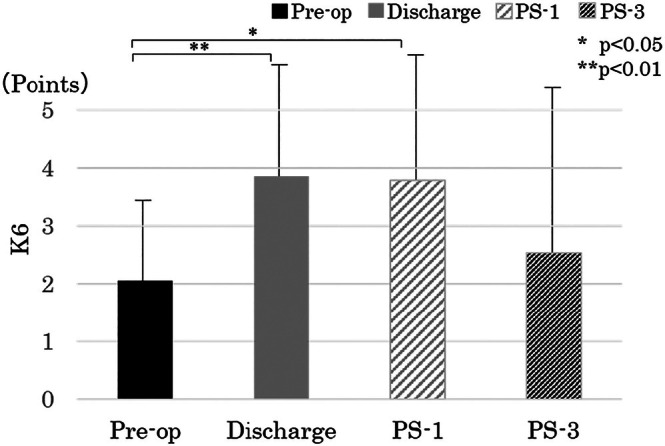
Changes in Kessler Psychological Distress Scale (K6) scores. A K6 score ≥ 13 indicates severe psychological distress. Preop = before surgery; PS‐1 = 1 month after surgery; PS‐3 = 3 months after surgery.

K6 scores increased transiently at discharge (*p* = 0.009) and PS‐1 (*p* = 0.012) but remained below the severe‐distress threshold (≤ 13) at all time‐points (Figure 6).

## 4 Discussion

Early postoperative UI after RARP has long been considered transient [19], yet its repercussions on QOL and work performance have seldom been tracked longitudinally. To our knowledge, this is the first prospective study to document the parallel trajectories of UI, presenteeism, and psychological distress from before surgery to PS‐3.

The present cohort showed marked UI severity and QOL deterioration in the first month, with gradual but incomplete recovery by 3 months. Concomitantly, work productivity (WHO‐HPQ) fell sharply at 1 month and rebounded only partially thereafter. The consistent negative correlations observed between UI indices and presenteeism across both follow‐ups underscore UI as a principal driver of early postoperative productivity loss.

### 4.1 Change in UI Over Time

Most previous studies have quantified male UI solely by pad counts. However, this method makes it difficult to appropriately evaluate mild UI using the number of urinary pads. Therefore, in this study, we used the Japanese version [20] of the ICIQ‐SF, which allows for a more detailed assessment. The ICIQ‐SF was developed by the International Consultation on Incontinence (ICI) and is a questionnaire whose reliability and validity have been verified, and a Japanese version [21] has also been developed.

A previous study on UI after prostate cancer surgery reported that 93% of patients were able to recover to a pad‐free state at 12‐months post‐surgery [22], while the median ICIQ‐SF total score in the early postoperative period was 0 points, 10 points at 1‐month post‐surgery, 7 points at 3‐months post‐surgery, 5 points at 6‐months post‐surgery, and 4 points at 12‐months post‐surgery. In this study, the ICIQ‐SF total scores were significantly higher from discharge to PS‐3 compared to those Preop, which was a similar trend to the previous study [23].

In particular, (1) the score was highest at PS‐1 (median 10 points), the time at which the patients' UI symptoms were most pronounced; and (2) although UI symptoms persisted up to PS‐3 (median 7 points), there was a tendency for them to improve over time.

These results suggest the importance of managing UI in the early postoperative period, and the need for proactive intervention, particularly at PS‐1. We also clarified the impact on patient QOL, which could not be captured by conventional evaluation methods based on the number of pads used.

### 4.2 Change in Presenteeism

The WHO‐HPQ [15] was established as an international assessment index for presenteeism and has a long history of use among researchers studying cancer patients [24]. The validity of the Japanese version has also been verified [25]. Existing studies on work ability after prostate cancer surgery have reported that in survivors less than 6 years after surgery, UI, postoperative adjuvant therapy, age, time since surgery, and comorbidities all affect work ability [26], and that 43% of patients responded that they were “unable to work at their pre‐diagnosis ability” 6 months after diagnosis [10]. However, these were all cross‐sectional studies and did not examine changes over time.

This study is the first to report on the longitudinal assessment of work productivity after RARP surgery from pre‐surgery to PS‐3, and is significant in that it clearly demonstrated a decrease in presenteeism. In this study, the WHO‐HPQ showed a substantial decline in work productivity, dropping from 81% Preop to 65% at PS‐1, and then recovering to 75% at PS‐3. In numerical terms, this represents a 16% absolute and 20% relative reduction in presenteeism at the point when most patients resume work. These findings underscore the need for perioperative interventions specifically addressing return‐to‐work challenges.

### 4.3 Relationship Between UI and Work Productivity

Previous reports have suggested that factors related to decreased presenteeism in prostate cancer survivors at 9–13 years after diagnosis include comorbidities, chemotherapy, and lack of support at work [11], but this is the first study to examine the relationship between early postoperative UI and presenteeism.

This study identified the severity of UI (ICIQ‐SF) and social limitations (KHQ) as factors related to decreased work productivity early after RARP. A significant negative correlation was observed between the severity of UI (ICIQ‐SF) and presenteeism at both PS‐1 and PS‐3, suggesting that the more severe UI, the lower the productivity. This is thought to be due to factors such as decreased concentration due to UI and interruptions to work due to changing incontinence pads. In fact, a meta‐analysis [27] on the work productivity of female workers reported that female workers with lower urinary tract disorders had significantly lower work productivity compared to women without lower urinary tract disorders, which is consistent with the results of this study.

In addition, social limitations (KHQ) showed a significant negative correlation with presenteeism and, at PS‐3, with a particularly strong impact on work and housework observed. At PS‐3, as activity returned and interactions with others increased, difficulties caused by UI were emphasized, which may have influenced the decrease in presenteeism. These findings indicate that both UI management and social activity support are important aspects of postoperative employment support. In terms of psychological impact, there was a low correlation between presenteeism and K6 (*r* = −0.26), but if PSA does not decrease approximately PS‐1 (PSA > 0.2), postoperative adjuvant hormone therapy or radiation therapy may be initiated, and it is possible that anxiety due to the possibility of recurrence may contribute to presenteeism. There was no significant difference in presenteeism between those who received adjuvant hormone therapy and those who did not. Side effects of hormone therapy include muscle loss and decreased physical strength due to decreased male hormones [28] and increased fatigue [29], so it is thought that this may affect presenteeism in the long term. The surveys in this study were conducted at PS‐1 and PS‐3, which is soon after the introduction of medication, and as this is a time when physiological muscle atrophy and anemia due to changes in the hormonal environment are not yet readily apparent, it is thought that there was no impact on presenteeism. Working style (normal work/employment restriction) and the time it takes to get to the toilet at work did not affect presenteeism. Postoperative employment restrictions provided a sense of security that “I can work according to my physical condition,” and did not affect presenteeism. Regarding the relationship between the time taken to get to the toilet in the workplace and presenteeism, as UI can be managed by using pads, the toilet environment (where to dispose of pads, where to change clothes) may have a greater impact than the time it takes to get to the toilet. Future research will need to further investigate the toilet environment in the workplace. On the other hand, when presenteeism was compared between regular and non‐regular employees at PS‐1, non‐regular employees had lower work productivity. The reasons for this include that there is no adequate system for granting and managing paid leave in place, and for those employed on a daily or hourly wage basis, 1 day of absence can directly affect living expenses. Thus, employees are unable to take time off for financial reasons, which may result in a decline in productivity. In the future, it will be necessary to investigate economic conditions, the number of paid vacation days, and company‐specific systems (such as whether or not sick leave is available).

### 4.4 Change in QOL

The KHQ was used to evaluate QOL associated with UI. The KHQ is an index that can comprehensively evaluate the personal and social aspects related to UI [30]. A previous study [31] compared the total unadjusted score of the KHQ before and after RARP surgery and reported that it was lower at PS‐1, after which QOL gradually improved, although it had not recovered to Preop scores by even 12 months post‐surgery. Furthermore, this is the first report to examine in detail the changes over time in each subscale of the KHQ after RARP and revealed more specific changes in QOL.

Compared to Preop, scores for all categories except for “Personal Relationships” were significantly decreased at PS‐1. The reason why no significant difference was observed for “Personal Relationships” is that the questions concerned life with a partner or spouse and life at home, and it is likely that the family support system was functioning adequately for many of the patients in our study. At PS‐3, the scores for “General Health Perceptions,” “Emotions,” and “Sleep/Energy” had improved to Preop levels. This is thought to be because, although UI was not completely cured, health and mental conditions had stabilized as patients became accustomed to its management. On the other hand, there was still a significant decrease in “Incontinence Impact,” “Role Limitations,” “Physical Limitations,” and “Social limitations” scores at PS‐3.

These results suggest that for patients who have returned to society by PS‐3, challenges remain in balancing UI management, such as discomfort associated with UI and pad changes, with social activity.

### 4.5 Change in Psychological Distress

It has been reported [32] that the risk of depression is high after radical prostatectomy for prostate cancer, and that this risk is further increased by radiation therapy and androgen deprivation therapy (ADT). However, there are only a limited number of longitudinal studies on mental state after prostate cancer surgery, and no reports have examined the changes in the early postoperative period in detail. In this study, evaluation was performed using the K6, which is simple to use and the validity of which has been established [33].

K6 scores showed a transient rise at discharge and at PS‐1 yet remained < 13 at all time‐points. Although severe anxiety or depressive states were uncommon, nearly one‐quarter of patients reported subthreshold distress (K6 ≥ 8) at PS‐1, indicating that a significant minority still require psychological attention. Integrating brief distress screening into routine follow‐up could therefore identify individuals who might benefit from early counseling.

### 4.6 Strengths and Limitations

Our results highlight a clinically meaningful, UI‐driven decline in work productivity that persists for at least 3 months RARP. Early pelvic‐floor rehabilitation, optimization of continence aids, and employer‐facilitated accommodations may mitigate presenteeism. Incorporating occupational therapists and industrial physicians into peri‐operative care could accelerate successful work reintegration.

This study has the following limitations. First, it is an observational study conducted at a single institution, and the results must be interpreted with caution before they can be generalized. Furthermore, the subjects were limited to working Japanese men, which limits the generalizability of results to groups with different cultural and social backgrounds and healthcare systems. Second, the evaluation was conducted for only a short period of time up to 3‐months post‐surgery, and the long‐term effects are unknown. In addition, as the survey was conducted by mail, the accuracy of the responses is limited, and the possibility of bias cannot be denied. Furthermore, the fact that no stratified analyses were conducted based on the subjects' age group or occupation may also affect the validity of the results.

Future issues include verification through multicenter and international collaborative studies and longer‐term follow‐up studies. Another important research topic is the development and verification of specific employment support programs for postoperative UI.

### 4.7 Clinical Implications and Conclusions

Our results highlight a clinically meaningful, UI‐driven decline in work productivity that persists for at least 3 months post‐RARP. Early pelvic‐floor rehabilitation, optimization of continence aids, and employer‐facilitated accommodations may mitigate presenteeism. Addressing UI proactively is therefore pivotal, not only for patient well‐being but also for sustaining workforce participation and reducing societal productivity loss after RARP.

## Author Contributions

N.N. was responsible for designing the study, collecting and analyzing the data, and writing the manuscript. T.T. was responsible for guiding the study design and reviewing the manuscript. A.K. was responsible for reviewing the final manuscript. All authors have read and agreed to the published version of the manuscript.

## Funding

The authors have nothing to report.

## Ethics Statement

The protocol was approved by the Teine Keijinkai Hospital ethics committee (No. 2‐020323‐00).

## Consent

All participants provided written informed consent.

## Conflicts of Interest

The authors declare no conflicts of interest.

## Data Availability

Research data are not shared.
